# A Deep Learning Approach for Molecular Classification Based on AFM Images

**DOI:** 10.3390/nano11071658

**Published:** 2021-06-24

**Authors:** Jaime Carracedo-Cosme, Carlos Romero-Muñiz, Rubén Pérez

**Affiliations:** 1 Quasar Science Resources S.L., Camino de las Ceudas 2, E-28232 Las Rozas de Madrid, Spain; jcarracedo@quasarsr.com; 2 Departamento de Física Teórica de la Materia Condensada, Universidad Autónoma de Madrid, E-28049 Madrid, Spain; 3 Department of Physical, Chemical and Natural Systems, Universidad Pablo de Olavide, Ctra. Utrera Km. 1, E-41013 Seville, Spain; crommun@upo.es; 4 Departamento de Física Aplicada I, Universidad de Sevilla, E-41012 Seville, Spain; 5 Condensed Matter Physics Center (IFIMAC), Universidad Autónoma de Madrid, E-28049 Madrid, Spain

**Keywords:** atomic force microscopy (AFM), deep learning, molecular recognition, variational autoencoder (VAE)

## Abstract

In spite of the unprecedented resolution provided by non-contact atomic force microscopy (AFM) with CO-functionalized and advances in the interpretation of the observed contrast, the unambiguous identification of molecular systems solely based on AFM images, without any prior information, remains an open problem. This work presents a first step towards the automatic classification of AFM experimental images by a deep learning model trained essentially with a theoretically generated dataset. We analyze the limitations of two standard models for pattern recognition when applied to AFM image classification and develop a model with the optimal depth to provide accurate results and to retain the ability to generalize. We show that a variational autoencoder (VAE) provides a very efficient way to incorporate, from very few experimental images, characteristic features into the training set that assure a high accuracy in the classification of both theoretical and experimental images.

## 1. Introduction

Atomic force microscopy (AFM) [1] in combination with dynamic operation modes [2,3] has become one of the key tools for imaging and manipulation of materials and biological systems at the nanoscale. It took almost a decade for one of these modes, frequency-modulation AFM, commonly known as non-contact (NCAFM), to fulfill the goal of achieving atomic or even subatomic resolution, as it was shown for the Si(111)-(7×7) reconstruction [4,5] or point defects and adsorbates on oxides [6]. The latest NCAFM breakthrough, the use of metal tips functionalized with a CO molecule at the tip apex, has provided access to the internal structure of molecules with totally unprecedented resolution [7,8]. These high-resolution (HR) AFM images have allowed molecular identification of complex organic compounds like the natural product breitfussin A, which results very difficult to characterize with other techniques [9]. The NCAFM ability to address individual molecules has paved the way for the identification of the intermediates (including radicals) and final products generated in on-surface reactions, shedding light into the formation processes and reaction pathways [10,11,12,13]. Moreover, NCAFM has been able to resolve more than a hundred different types of molecules in one of the most complex and economically relevant mixtures that exist: asphaltenes, the solid component of crude oil [14].

The main contrast mechanism for AFM with inert tips is Pauli repulsion [7]. This repulsive force contribution arises because the electron densities of tip and sample overlap, resulting in increasing frequency shift—changes in the oscillation frequency of the cantilever holding the tip due to the tip-sample interaction—that are observed as bright features in the constant height AFM images above atom positions and bonds, reflecting the molecular structure. Increasingly accurate AFM simulation models [15,16,17,18,19] have been developed to explain the observed image contrast in terms of the different contributions to the tip-sample interaction. They have contributed to elucidate the influence of the electrostatic force [20,21], the role of the CO-metal tip charge distribution [19,22], and the interplay of the short-range chemical interaction and electrostatics in bond order discrimination and the imaging of intermolecular bonds [23]. In spite of the wealth of information provided by NCAFM experiments and these advances in the interpretation of the observed contrast, the unambiguous identification of molecular systems solely based on NCAFM images, without any prior information, remains an open problem.

Artificial intelligence (AI) techniques, also known as machine learning (ML), have demonstrated an extraordinary ability to differentiate patterns and perform predictions with high accuracy in many different fields [24,25,26,27]. Recent innovations in GPU technology have enabled the development of computer vision techniques [28,29,30,31]. These technological breakthroughs supported the design and training of complex, multilayer convolutional neural networks (CNN). Deep learning, based on the use of CNNs, has opened up the prospect of providing machines with capabilities hitherto unique to human beings [32,33,34,35]. CNNs have indeed exceeded the early expectations, surpassing the human capacity in tasks such as the identification of the content of photographs performed in the ImageNet 2012 Challenge, where the ResNet-152 model achieves a 3.57% top-5 error, while the human one is 5%.

The main challenge in AI is to determine the complexity of the model (the number of layers and the filters included in each layer) needed to strike the right balance between the specialization needed for an accurate prediction [36,37,38] and the ability to generalize [39]. A predictive model must contain only the required number of parameters (and no more) than strictly necessary to perform the prediction. If the model has excessive degrees of freedom for the complexity of the dataset, we run into overfitting, a well-known problem in deep learning [38,40]. In overfitting, the model has acquired the ability to classify with very high accuracy a limited set of data, but it is not able to generalize to slightly different data of the same class [41,42]. The problem of overfitting arises naturally in the application of deep learning to image recognition because images have to be represented in high dimensional spaces (one dimension for each pixel in the image) [40,41]. The amount of data needed to adjust the model parameters increases significantly with the space dimension. Even with large training datasets, the model is so complex that we will be forced to show the same data several times (training epochs) to the model in order to adjust its parameters. The consequence is that the model will specialize in training data but will be unable to generalize.

Together with the technical problems described above, the possible application of deep learning to the problem of molecular identification based on AFM images has to face two main challenges that are intrinsic to the technique: how to achieve chemical identification within the molecule at the single atom level, and how to deal with markedly non-planar, 3D structures. The last problem has been recently assessed by a combination of AFM experiments and image simulations with machine learning in order to determine partial 3D structures of small molecules [43]. The strategy is based on a neural network that converts a stack of experimental constant-height AFM images taken at different tip-sample distances into a generated “height map” that gives information on atom positions within the molecule. Regarding single-atom identification, AFM chemical sensitivity has been demonstrated for a few elements incorporated in planar semiconductor surfaces using the maximum attractive force [44]. This attractive force regime is not accessible with CO and other inert tips that experience a repulsive interaction with molecules. Other possible strategies based purely on force spectroscopy are challenged by the fact that, at the tip-sample distances explored in these experiments, the charge distribution on atoms of the same element is different depending on their local bonding environment and the global molecular stoichiometry [23]. Elemental sensitivity can be boosted by identifying features in the 2D images and 3D force maps that reflect the highly anisotropic spatial decay of the molecular charge density and provide a way toward elemental identification [23]. For example, the replacement of C–H groups by N atoms in a benzene molecule modifies the charge density and locally distorts the image, with sharper vertices around the N atoms, making the N distinct from the C atoms. On the other hand, linear features have been linked with halogen atoms [9,23]. The ability of models based on CNNs to recognize these features on AFM images has not been explored yet.

The goal of this work is to fill this gap with the development of a strategy to combine high-resolution AFM imaging and machine learning in order to achieve molecular identification. We focus on a set of quasi-planar molecules that spans relevant structural and compositional moieties in organic chemistry. We describe how, from each of these molecules, we build the necessary training dataset of 2D theoretical images, striking the right balance to incorporate enough variation and to prevent overfitting.

Firstly, we show the limited performance of two well-established deep learning models for image recognition [45,46] in their application to molecular classification based on AFM theoretical images. Their limitations can be understood by analyzing the transfer of relevant information across the different layers in the model. Based on this analysis, we develop a specific architecture for molecular identification that shows an excellent performance in its application to theoretical images. Finally, we test the model, trained exclusively with theoretical images, with experimental images [47], trying to understand the differences between experimental and simulated AFM images that hamper a proper classification. We solve this problem with a variational autoencoder (VAE) [48,49] that allows us to generate, from very few experimental results, a small set of images that incorporate some of their characteristic features. The addition of this small set to the training process of our model leads to an optimal identification.

## 2. Materials and Methods

### 2.1. The SPMTH-60 Dataset of AFM Images

**Figure 1:**
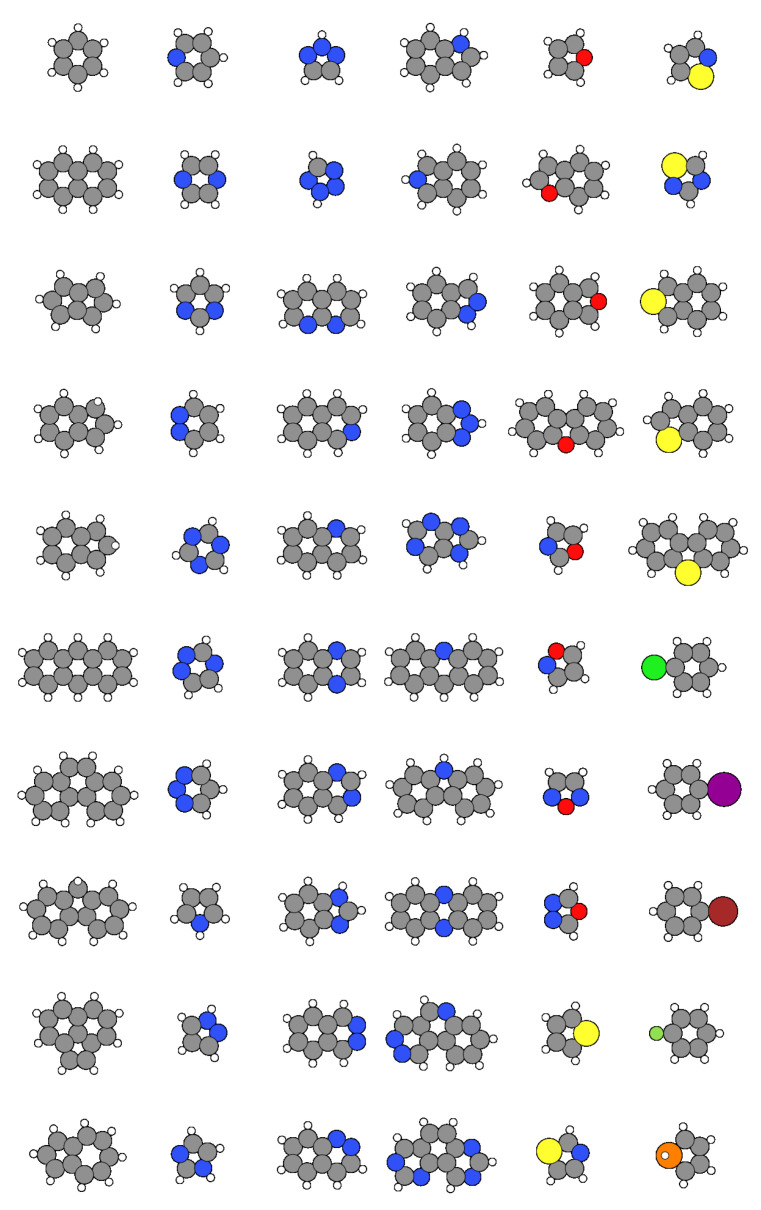
Ball and stick representation of all the molecules included in the SPMTH-60 dataset. Carbon (grey), hydrogen (white), nitrogen (blue), oxygen (red), sulphur (yellow), chlorine (lime), iodine (purple), bromine (maroon), fluorine (green), and phosphorus (orange) atoms are represented by color balls.

**Figure 2:**
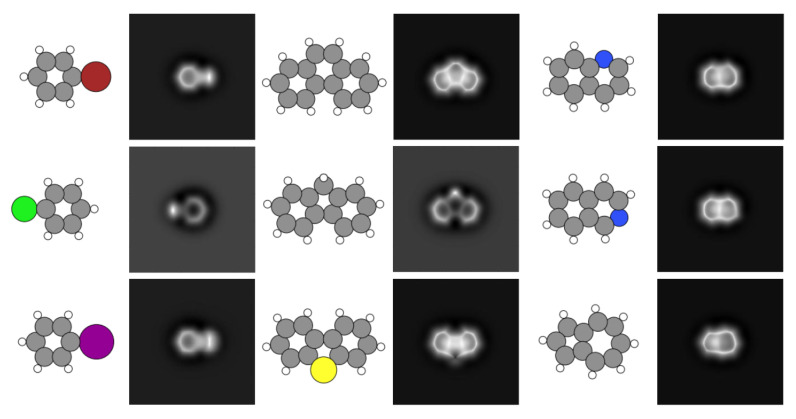
Theoretically simulated AFM images for different molecules. Each column displays three molecular structures that give rise to very similar AFM images, making it extremely difficult for a human to identify them. Atoms are represented with the same color code used in Figure 1.

The SPMTH-60 dataset mostly contains a collection of theoretical constant-height AFM images built from a selection of 60 essentially flat organic molecules (see Figure 1) whose structures were obtained from the *PubChem* web [50]. These molecules are mainly benzene derivatives, including up to three hexagonal and pentagonal rings with planar structure. The different classes of molecules represented in the dataset include 10 different atomic species (C, H, N, P, O, S, F, Cl, Br, I), as shown in Figure 2. These molecules are chemically grouped in polycyclic aromatic hydrocarbons (including benzene), simple heterocycles, combinations of hydrocarbon cycles with the heterocycles and other aromatic derivatives like halides. Although relatively small, this set includes some of the most common structures and relevant chemical species in organic chemistry and poses some of the fundamental challenges in molecular classification, like the discrimination among the different halides in the same molecular structure.

**Figure 3:**
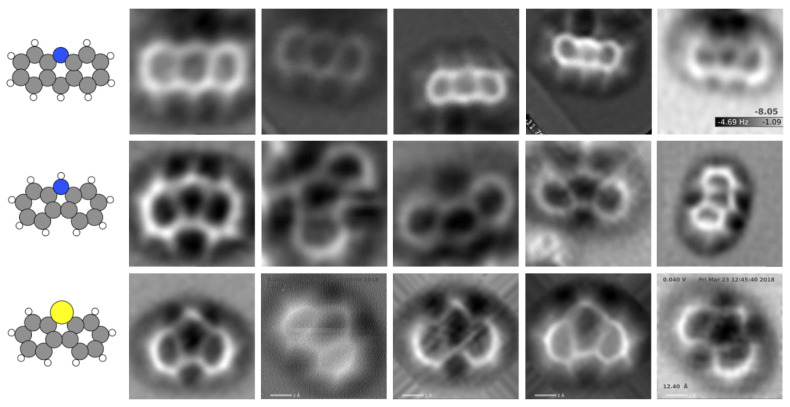
Experimental images for acridine, carbazole, and dibenzothiophene [47,53]. Each row shows, for the same molecule, the large variability introduced in the AFM images by different experimental conditions. This variability represents a challenge for molecular classification based on AFM. The three images in the first column were reprinted with permission from ref. [47]. Coppyright 2019 American Chemical Society. The rest of the images have been taken by Drs. P. Zahl and Y. Zhang during their work [47] and kindly provided to us [53].

AFM experiments are conducted on adsorbed molecules, whose structure and electronic properties may be affected by the interaction with the substrate. These changes, together with different experimental conditions, lead to a significant variability in the recorded AFM images, as shown in Figure 3 for three of the molecules considered in our dataset: acridine, carbazole, and dibenzothiophene. In order to take into account these effects, SPMTH-60 includes a set of images generated for each molecule containing 48 different configurations, that mimic the effect of the adsorption on the molecular structure. In addition, we include simulations 168 different sets of AFM operation parameters for each configuration (see Section 2.2 for details). Thus, the total number of simulated images for each molecule is 8064 and they are generated in a 224×224 pixels format suitable for the standard image recognition models discussed below.

Most SPMTH-60 dataset simulations are calculated with a simplified version of the full-density-based model (FDBM) [19,23] implemented in the latest release [51] of the probe particle model (PPM) [16,52]. The details of this implementation are discussed in Section 2.3. As described below, the FDBM model has been used for additional AFM simulations for certain molecules in the dataset. The tip and sample electronic charge densities and the sample electrostatic potential needed for the simulations with either the PPM or the FDBM methods have been calculated using quantum first-principles calculations, using molecular structures taken from the *PubChem* web-supported database of chemical compounds [50]. More details about the AFM simulations and first-principles calculations can be found in following subsections.

As discussed below, machine learning models trained exclusively with theoretical images completely failed in the classification of experimental images. We have augmented our training data set with 540 images generated with a VAE from three experimental images for acridine, carbazole, and dibenzothiophene taken from Reference [47] (see Figure 3). An additional set of 110 unpublished images [53]—68 for acridine, 11 for carbazole, and 31 for dibenzothiophene—have been used for testing the different classification models. Some of these images are also shown in Figure 3.

### 2.2. Molecular Orientations and Operation Parameters for AFM Simulations

AFM experiments are conducted on adsorbed molecules, whose structure and electronic properties may be affected by the interaction with the substrate. In order to take into account these effects, SPMTH-60 includes a set of images for each molecule generated with different molecular orientations, that mimic the possible effect of the adsorption. In particular, we have applied 48 rotations arising from the combination of the following Euler angles: α={0,20,40}, β={0,1,2,3}, γ={0,3,6,9} degrees. The *z*-axis is perpendicular to the molecular plane, and α defines the rotation around this axis, while the angles β and γ correspond to rotations around the two mobile axis that control the motion of the molecule out of the original molecular plane. We use small values for β and γ in order to mimic the adsorption configurations found when these molecules are deposited on substrates, like Au, used in the real experiments. Since all the molecules considered here are flat and possess some symmetry elements this choice with only 48 rotations is enough to properly reproduce the usual experimental situations.

To ensure that SPMTH-60 reflects all the image variants of each molecule, we have considered a variety of operational AFM settings for each of the rotated configurations described above. As a result, a group of AFM images is provided for each of the molecules. Namely, we have simulated each structure with 4 different values of the elastic constant describing the tilting of the CO molecule (0.40,0.60,0.80,1.00 N m${}^{-1}$), 6 different oscillation amplitudes of the cantilever (0.40,0.60,0.80,1.00,1.20,1.40 Å), and 7 different tip-molecule distances of closest approach (2.80,2.90,3.00,3.10,3.20,3.30,3.40 Å). Consequently, SPMTH-60 consists of 48×168=8064 AFM simulations for each of the 60 molecules, resulting in a total of 483,840 images with resolution 224×224 pixels. We have trained the models in this paper by splitting the dataset into training, validation, and test sets with 314.460, 120.960, 48.420 images, respectively, where all the molecules are equally represented (same number of images) in each of these subsets.

### 2.3. AFM Simulations with the Approximate Version of the FDBM Model Implemented in the PPM Suite of Codes

For the implementation of the FDBM model, the latest release of the PPM calculates the electrostatic force field as a convolution of the neutral tip charge density—the difference between the total density of the molecule and sum of the atomic densities of the C and O atoms—and the electrostatic potential of the sample. In previous versions, a quadrupole term was used to describe the electronic charge distribution of the CO molecule acting as the tip [52]. On the other hand, the short-range chemical interaction is calculated as a convolution of the electronic charge density of the sample with the total charge density of the CO molecule [23], using a value of the exponent α=1 in the convolution. Finally, van der Waals forces are approximated by the attractive part of the Lennard-Jones potentials. The approach of the tip to the sample is performed in steps of Δz=0.1 Å and the position of the tip is relaxed in each step [52].

### 2.4. First-Principles Calculations

The simulations for both the electronic charge density and electrostatic potential of each structure and the charge density of the CO molecule acting as the tip were are based on density functional theory (DFT) following the implementation provided in the VASP code [54,55]. An energy cut-off for the plane-wave basis set of 400 eV was used in combination with pseudopotentials constructed after the PAW method [56,57]. The Perdew–Burke–Ernzerhof functional [58] was chosen to reproduce the electronic exchange and correlation, supplemented by the D3 semi-empirical correction to account for the dispersion interactions [59].

The bare molecules were subjected to single-point calculations (electronic self-consistency) using the geometries provided in the *PubChem* web [50]. In few cases we performed a full relaxation of the molecules on a Au(111) rectangular substrate containing three layers and a total of 108 gold atoms. In all cases, the VASP outputs were rewritten into xsf format with the xsfConvert modular code in order to use them in the PPM code.

## 3. Results and Discussion

### 3.1. Standard Deep-Learning Models for Image Classification

The idea of providing a machine with the ability to classify images is one of the main challenges in machine learning and has fostered a significant amount of work in recent years. Deep learning techniques, in particular convolutional neural networks (CNN) architectures, have played a key role in this effort and several models based on this paradigm have achieved remarkable results. We have applied two of these models, MobileNetV2 [45] and VGG16 [46], to our dataset in order to verify its performance in the task of molecular classification based on AFM images. See details Appendix A for the implementation and training of the models.

#### 3.1.1. MobileNetV2

MobileNetV2 [45] is an extremely deep architecture with a very large number of blocks, each composed of multiple layers, that include a large amount of filters. This complexity pays off and truly outstanding results are obtained in different classification tasks. There are two key quantities to determine the performance of the model: the loss and accuracy metrics. Accuracy represents the fraction of the images that were correctly classified. The loss function estimates the model error at each iteration of the optimization process comparing the predictions of the network and the true target. This information is used to update the weights in order to reduce the error in each evaluation. We use as loss function a multi-class cross-entropy loss, which is the preferred option under the inference framework of maximum likelihood [60]. When MobileNetV2 is applied to the SPMTH-60 dataset, the evolution of the loss and accuracy metrics as a function of the epochs of training and validation (see Figure A2) shows that the model quickly runs into overfitting, reflecting that it has excessive degrees of freedom to address this classification.

In order to avoid overfitting, we have employed different common procedures: we have halved the number of filters in each layer, applied a strong image data augmentation to the training set (see Appendix A.1 for a description of the augmentation strategies), and stopped the training at a very early stage (at epoch 17, see Appendix A.2 for details) [61]. With this strategy, MobileNetV2 reaches a high accuracy in the classification of purely theoretical images. However, as shown in Table 1 and discussed in more detail in Section 3.3, it completely fails with experimental images of some of the molecules included in the dataset. Given the flexibility provided by the large number of parameters and its proven efficiency in image classification tasks with other image datasets, our results indicate that this model is not suitable for the classification of AFM images of SPMTH-60.

#### 3.1.2. VGG16

**Figure 4:**
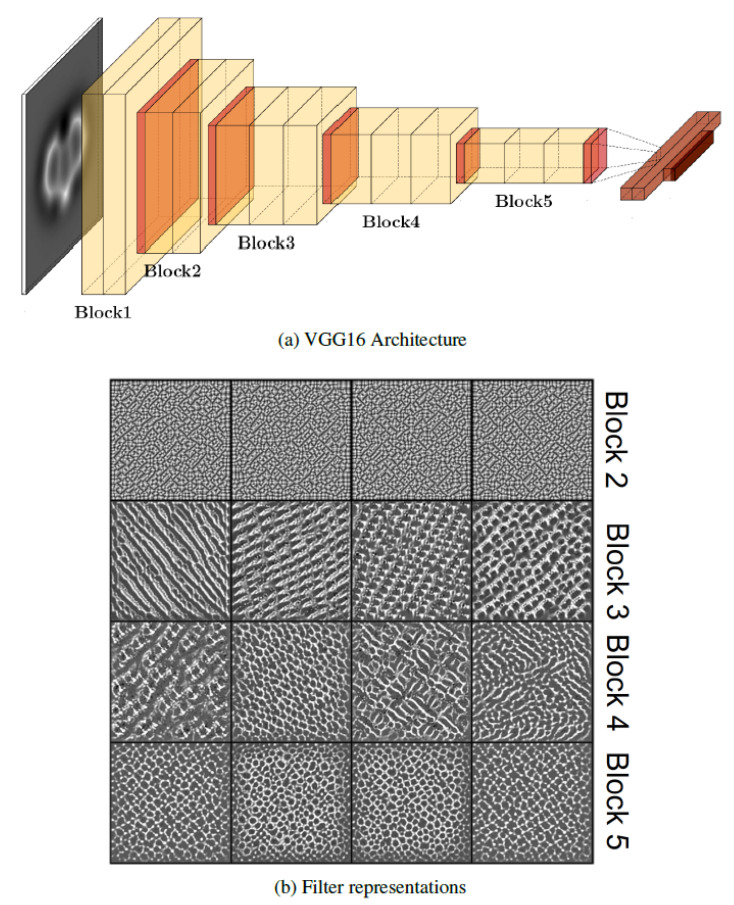
(**a**) The VGG16 architecture combines five blocks, each composed by a series of convolution (yellow), pool (red), flatten (purple), and fully connected (brown) layers, in order to reduce the initial input, a 224×224 image, into a probability vector with the size of the number of different classes (see ref. [46] for a definition of these layers). (**b**) Representations of the filter patterns learned by some of the convolutional layers of the VGG16 model during the training with SPMTH-60 dataset. From top to bottom, each row includes four filters located in the last convolutional layer of blocks 2, 3, 4, 5, respectively. Although the patterns in blocks 4 and 5 show specific features in the AFM images such as ring deformations, those on block 3 display non-specific features that are usually learned in the first layers of the model. Only random patterns can be observed in the last layer of block 2. Since the filters are modified by applying back propagation, none of the filters belonging to the first two blocks have been updated during the training, a clear indication that the model is in overfitting.

Learning from the results obtained with MobileNetv2, we have applied the VGG16 architecture [46] to the SPMTH-60 dataset. This is a much simpler model than MobileNetV2 (see Figure 4), developed specifically to treat $224^{2}$-pixel images and sequentially composed of five convolutional blocks with multiple 3×3 kernel-sized filters (see Figure A4 for examples) and one fully connected block ending in a probability vector (of length equal to the number of classes) that relates each vector component to a class defined in the dataset.

**Figure A1:**
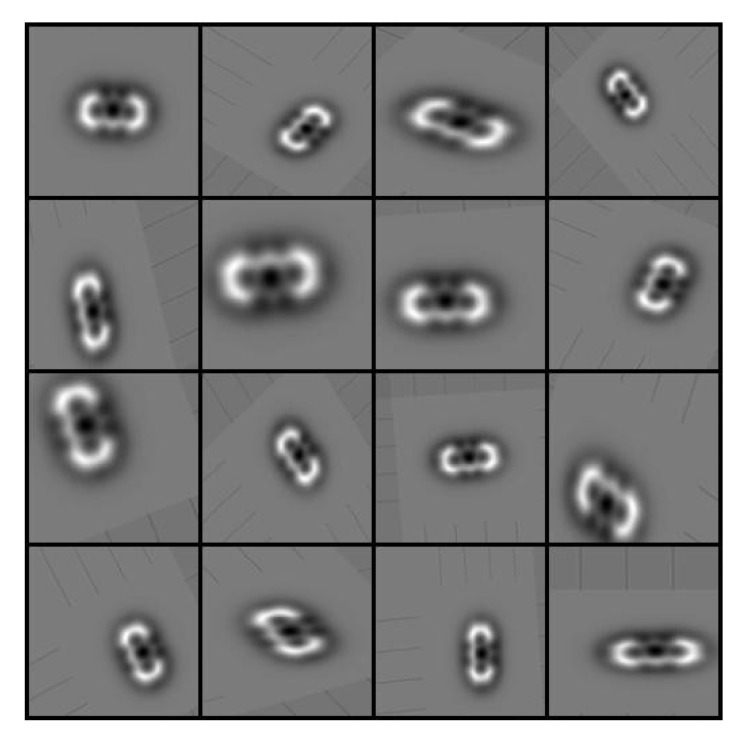
Top-left image is a theoretical simulation of the AFM image of acridine calculated with the approximate version of the FDBM model implemented in the latest release of the PPM. The remaining images are examples of random deformations of this theoretical AFM image generated by the image data generator (IDG). These deformations can include rotations, flip, zoom, shear, and shift of the image, or a combination of them. The selection of the deformation parameters is randomized for each theoretical image at each training epoch in order to add variation to the data used for the training and avoid overfitting.

**Figure A2:**
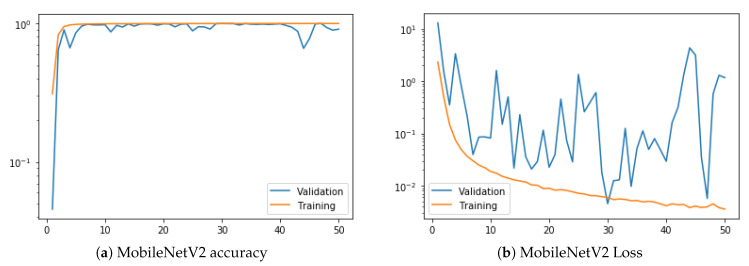
Loss and accuracy metrics of the MobileNetV2 model (with halved filters in each layer) during the training. The metrics of the data with which the model is fitted (training) are shown in orange while the blue graph shows the plots of the validation set, used as a guide to adjust the hyperparameters of the model. The metrics indicate that the model achieves high accuracy in that is too complex for our training dataset, leading it to overfitting.

**Figure A3:**
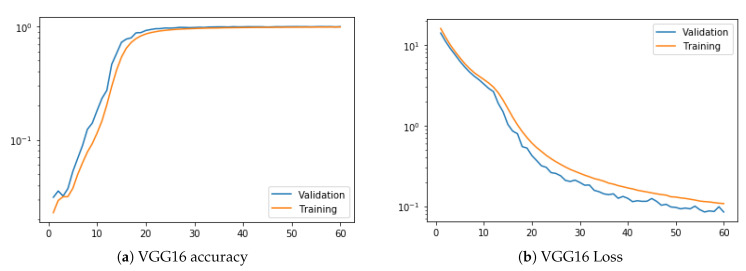
Performance of the loss and accuracy metrics of the training (orange) and validation (blue) data during the training of the VGG16 model. The values of the metrics are suitable, however in the main text we have seen that the model does not generalize to experimental images, which indicates that loss and accuracy metrics during training often cannot be taken as the only method of evaluating a model.

In the VGG16 training with SPMTH-60, it has been necessary, as in the case of MobileNetV2, to increase the image features of the training set by applying an image data augmentation (Figure A1) in order to prevent overfitting. Under these conditions, the loss and accuracy metrics behave reasonably well (see Figure A3), reaching, after 60 epochs of training, an 0.99 accuracy with the testing set. However, when confronted with experimental images, we obtained another dramatic failure (see Table 1), indicating that this model has also over-fitted.

**Figure A4:**
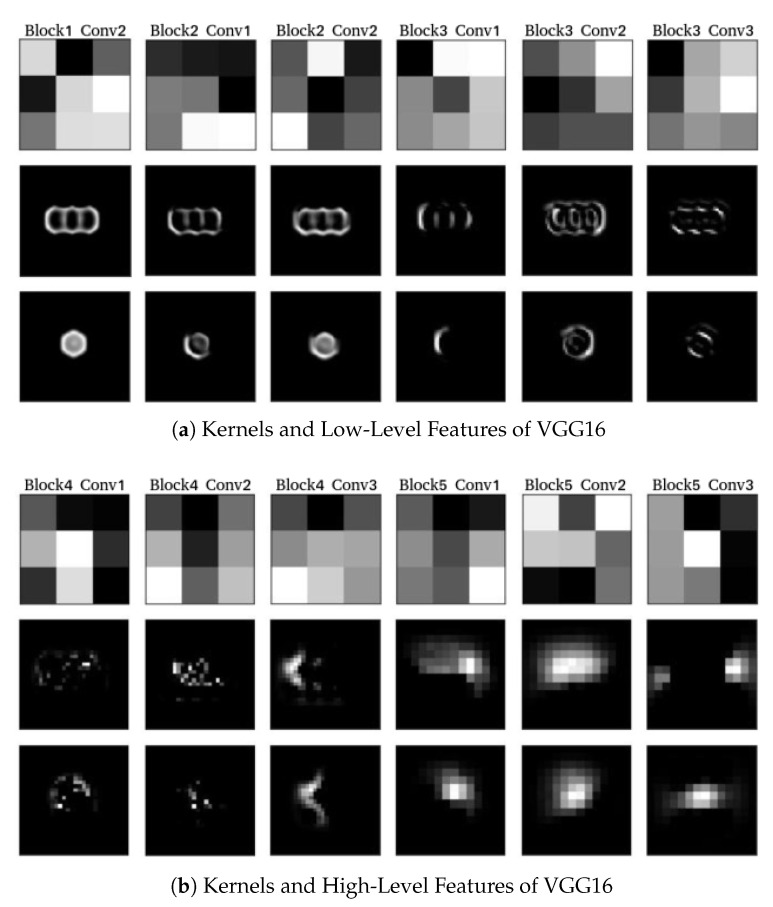
Kernels of VGG16 and the respective activations of the AFM images of anthracene and benzene molecules. From left to right the activations represent features from lowest to highest level. The activations show the matches of the kernels with the input image of the respective layer, which is the information that *“passes through”* that filter to the next layer. The filters are not more complex in deeper layers, reflecting that the features are of a higher level when the image has been processed by more layers.

In order to understand the performance of VGG16 in the AFM image classification, we carried out a visual representation of the learning achieved by different filters located in different blocks. Figure 4b shows in each row four filter patterns included in the last convolutional layer of blocks 2 to 5 in the VGG16 architecture. Each of these filter patterns represents an image that maximizes the activation of a selected filter. A filter is activated when its associated kernel—a defined pattern in a small group of neighboring pixels designed to capture some specific characteristic of the images (see Figure A4 for some common examples and Reference [60] for a detailed description)—matches some part of the image that the filter is processing. Our initial guess for the input image is simply a grayscale image built from random noise. Then, we applied an iterative procedure (based on the gradient ascent [60]), to modify the input image in order to maximize the activation of a particular filter. The images shown in Figure 4b are the final output of this process.

At this point, we should recall the basics of the back propagation algorithm used in the training of deep learning architectures. During training, the model performs a prediction resulting in a probability vector. If this prediction is not equal to the target label, this error is propagated from the output layer (the last layer in the model) backwards. The error propagation method calculates the gradients of the loss function computing the chain rule for derivatives to determine the contribution of each neuron in the previous layer to the error and to modify its parameters accordingly. Considering that the depth of a deep learning model like VGG16 is 16 layers (13 convolutional and 3 fully connected layers, Figure 4a, this backpropagation algorithm mostly modifies the deepest layers (those closer to the output layer) while the influence of the first layers on the error reduction is very slight. This is the key to understand why the first layers learn low-level features and their filters remain generic, while the deepest layers specialize in high-level features (See Figure A4).

These ideas are the key to understand the results shown in Figure 4b. Looking at the last two rows, the patterns corresponding to blocks 4 and 5 show specific features in the AFM images, such as ring deformations. The patterns associated with the last convolution of the third block display non-specific features that are usually learned in the first layers of the model. The filters of the last layer of the second block show random patterns, a clear indication that they have not been updated. Since the filters are modified by applying backpropagation, it follows that none of the filters belonging to the first or second blocks have been updated during the training process. These results conclusively prove that, despite having applied an image data augmentation to the training set, the VGG16 architecture contains more parameters than those that strictly required to perform the prediction. This scenario is a clear indication of overfitting.

In summary, we have shown that two of the best standard models for automatic image classification do not perform well with the SPMTH-60 dataset and lead to overfitting. Among the reasons for their limited performance, we can point out the following: Both models have been tested with larger datasets composed by color images (with three different channels to represent the color, RGB or any of the alternatives), while AFM data, and correspondingly SPMTH-60, is composed by grayscale (single channel) images. Secondly, standard datasets contain hundreds of classes while the SPMTH-60 dataset is composed by only 60 different classes. Finally, AFM images share rather similar features, i.e., the basic features in the AFM image of a benzene are not substantially different from the ones for anthracene, and this also applies when comparing pyridine and pyrimidine. In the task of discriminating between these images is not so important that different filters are activated but that the same filters are activated in different areas of the image (See Figure A4).

### 3.2. Our ML-AFM Model

**Figure 5:**
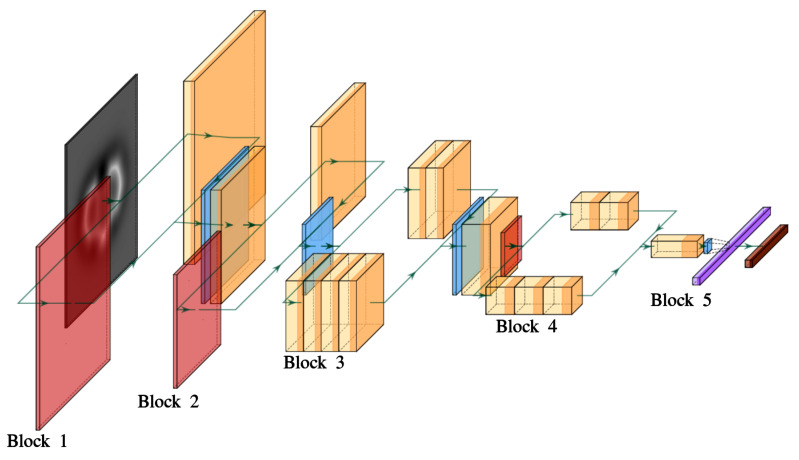
ML-AFM model architecture designed to capture the characteristics of AFM images and to prevent a too specialized training (overfitting) in AFM image classification. The model combines five blocks, each of them with a series of different layers such as convolution (yellow), pool (red), dropout (blue), flatten (purple), and fully connected (brown) layers. Table A1 provides a detailed description of each layer, including the number and characteristics of all the filters.

The analysis carried out for MobileNetV2 and VGG16 models shows that standard architectures for image classification run into over-fitting before enough features are learned and generalized to classify AFM experimental images. We have developed a specific machine learning model, the ML-AFM model (Figure 5) for this classification. The model includes convolution, pool, dropout, flatten, and fully connected layers as VGG16, but is designed with particular emphasis on preventing overfitting by a combination of different strategies, such as (i) an optimal number of convolutional layers and filters and the use of concatenation between them in order to introduce alternative *paths*—different ways provided by the model in order to link the input and output layers of the model—(ii) the presence of dropout layers, and (iii) the regularization in convolutional layers (See Appendix A.4). Table A1 provides a complete description of each layer in the model.

In order to prevent overfitting, the first two blocks of our model (Figure 5) provide branches through either a pool layer or a series of convolutional layers. The same goal motivates the presence of the branches in the third and fourth blocks, where each branch has a different depth, and, thus, can fit their filters to specialized or to general features. Deep learning models need enough depth—the number of convolutional layers between the input and output for a given path—to specialize in the classification. According to our analysis of the performance of VGG16 (see Figure 4), a minimum of nine (and a maximum of 12) convolutional layers were updated during the VGG16 training. Therefore, we have implemented model paths with a depth of twelve convolutional layers (see Figure 5). Each convolutional layer contains several filters and each filter specializes its kernel in a particular kind of detection. Since we are dealing with grayscale images, and AFM images shared rather similar patterns, our model has few filters in each layer (Table A1) compared with the standard classification models [45,46] developed to perform color (three-channel) image classification.

The goal of a dropout layer is to turn off randomly chosen neurons at each epoch during the training. This technique ensures that the model does not assign a specific path for each input, making the model robust and preventing overfitting [41]. We have located these layers at the beginning of each block, a place in the model common to all of the paths and where, therefore, all information converges.

Finally, we introduce a strong regularization in the ML-AFM model. We have added regularization kernels (using the L2-norm) to some key layers of the model (See Table A1). The purpose of these kernels is similar to the one described for the dropout layers, but they act in a different way: they introduce regularization by penalizing the layer parameters during backpropagation by incorporating errors into the loss function that optimizes the network.

**Figure A5:**
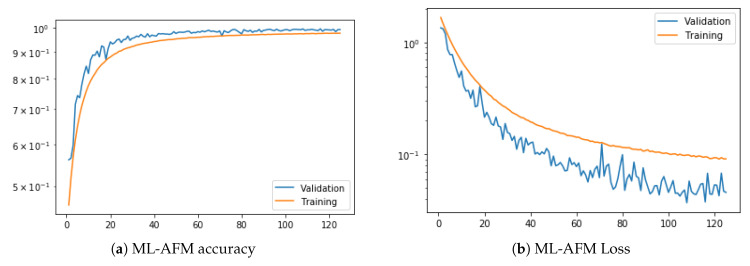
Loss and accuracy metrics of the ML-AFM model during training. The evaluation on the training data is shown in orange while the validation data are represented by the blue graph. The accuracy on validation data is better than on training data because the IDG is applied only to the training set. It should be noted that the effort made to prevent overfitting is reflected in the fact that the accuracy loss function has a slower growth than in the VGG16 and MobileNetV2 trainings. As a consequence, the model needs more epochs to adjust its weights but the training does not result in a too specialized model that is able to generalize.

The training of the model includes, as for the standard models, an image data augmentation (Figure A1), and is performed with an Adam optimizer [62]. The training is carried out up to 130 epochs. The loss and accuracy metrics for the training and validation are shown in Figure A5.

The prediction of the ML-AFM model with the theoretical test set is as accurate as the one obtained with the standard models (0.99 accuracy). However, when considering the performance with the set of experimental images described at the end of Section 2.1 (see Figure 3), while the standard classification models fail dramatically, our model retains a reasonable accuracy, between 0.45 and 0.82 depending on the molecule considered (see Table 1). The implications of these results are clear: The standard models cannot generalize the classification. They have overspecialized in the clean features displayed by theoretical images and fail completely when confronted with the noisier experimental images. On the other hand, our ML-AFM model achieves acceptable results, with the same training based only on theoretical simulations, due to the emphasis placed in its design to prevent overfitting.

### 3.3. A Variational Autoencoder (VAE) to Improve the Classification of Experimental AFM Images

The limitations of our ML-AFM model in the classification of experimental AFM images discussed above have prompted us to explore different ways to improve our training set. It is clear that experimental images have some characteristic features that are not captured by simulations. Differences between experimental and theoretical AFM images for a given molecule could be due to the approximations made in the simulation of AFM images, or to the fact that molecules relax and deform due to the interaction with the substrate, while we are considering ideal, gas-phase structures in the simulations. In order to cope with the first issue, 36 AFM images calculated using more sophisticated simulation methods [19,23] have been added to our training set for each of the three molecules in the experimental test. They represent different AFM operation conditions: six tip heights, three oscillation amplitudes and two different values for the CO tilting stiffness. We have also included simulated images of the same molecules adsorbed on Au(111) after a full relaxation using DFT. None of these attempts have resulted in a substantial improvement in the classification of the experimental images.

**Figure 6:**
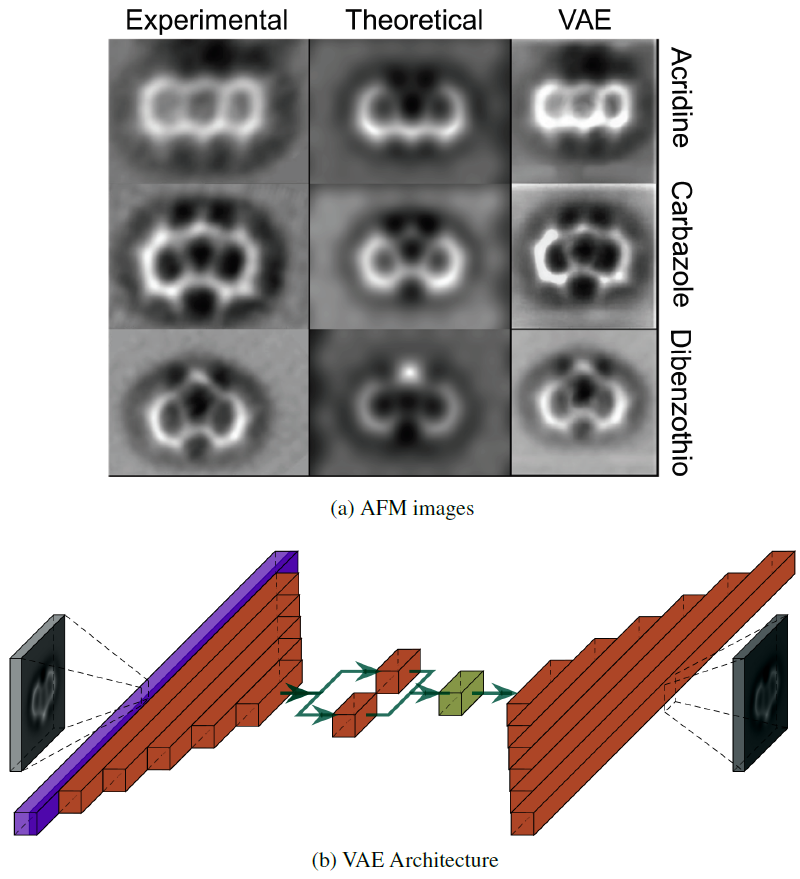
(**a**) Images of (from top to bottom) acridine, carbazole, and dibenzothiophene. From left to right, each image corresponds with the experimental AFM image used for the projection into the latent space, the theoretical simulation, and one of the images generated by the VAE. (**b**) VAE architecture, showing the flatten (purple), fully connected (brown), and the lamdba (khaki) layers. The lambda layer represents the latent space, where noise from a normal distribution is added to the projection produced by the encoder in order to generate, through the decoder, a new image that include features characteristic of the experimental images.

In what follows, we discuss how to augment our training data set including images with features similar to those found in the experiment generated with a variational autoencoder (VAE) [48,49] in order to improve significantly the performance. An autoencoder is an unsupervised neural network composed by two neural networks called encoder (input compression) and decoder (decompression). The encoder produces a representation of the input data in a low dimensional vectorial space (code space). The decoder uses a point of the code space (compressed representation) as input and generates an image as close as possible to the encoder input. Our implementation (see Figure 6b) uses a variant called *variational* autoencoder that adds a probabilistic contribution to the code space turning it into a latent space (a code space with probabilistic distributions).

Autoencoders have been used for different purposes such as image denoising [63], image segmentation [64], reconstruction of deleted areas of images [65] and data augmentation [66]. In our case, the goal of the VAE is to incorporate characteristic features of the experimental images to produce new elements for our training set. The encoder not only represents each input image as a compressed representation in the latent space, a three-dimensional (3D) vector in our case, but also clusterizes it attending to similar image features, distributing the inputs of each class in the same area of the latent space. Then, each point of the latent space can be used as input for the decoder, that produces a reconstruction of the input image. (See Figure 6). In order to add experimental features to the training set, we project an experimental image for a given molecule into a compressed representation (a 3D vector) in our latent space. Adding a small noise ϵ from a normal distribution to each of the components of this vector (Figure 6b), we generate new points around the representation. Feeding the decoder with one of these points, we obtain new images for that molecule, that retain the characteristic features of the experimental images, and can be incorporated into the training of the classification model. Details of the dimensions of the VAE layers and training can be found in Appendix A.5.

The VAE has to be trained with theoretical images. Our results show that, in order to endow the VAE with the ability to reflect the characteristic features of the experimental AFM images, it is necessary to ensure that the training images reflect details that are absent from the the simulations of the isolated molecules. Simulated images for the relaxed structures obtained with DFT for these molecules upon adsorption on Au(111) slab have been used to replace the images obtained from the isolated structures. In addition, we have applied an augmentation using the image data generator (IDG) (see Appendix A.1 for details) during the training of the VAE. To enable the autoencoder to learn the deformations applied, these deformations must be identical for the input and output. The combined effect of these two techniques enables the model to perform an accurate and robust reconstruction of the experimental images (Figure 6a).

We have applied the encoder to three experimental AFM images, one for each of the molecules acridine, carbazole, and dibenzothiophene (left column of Figure 6a), and save their representation in the latent space. Then, we randomize the selection of 180 points around each of this representation following a normal distribution. Finally, we use each of these points as an input for the decoder network, generating new images. In this way, we incorporate 3×180=540 new images into their respective classes in the training subset of the SPMTH-60 dataset, up to a total of 315.036 images. We have retrained the two standard classification models, MobileNetV2 and VGG16, and our own ML-AFM model with this slightly extended training set. Their performance in the classification of experimental images is shown in Table 1. Standard models improve significantly their performance, but they are still limited due to overfitting. Meanwhile, ML-AFM is able to almost recover the accuracy achieved in the classification of theoretical images. We have accomplished this feat with the addition of just 0.17% experimental-like images, generated from only three real experimental images through our VAE, to the training dataset.

## 4. Conclusions

Our results show the potential of deep learning models trained with theoretical simulations for a classification of molecular species based on constant height AFM images. To this end, we have developed the SPMTH-60 dataset, generated from a selection of 60 essentially flat organic molecules that includes some of the most common structures and relevant chemical species in organic chemistry. Considering, for each of these molecules, 48 different molecular orientations to capture the possible effect of adsorption and 168 combinations of AFM operation parameters (average tip height, cantilever oscillation amplitude and CO tilting stiffness), we have built a total dataset of almost half a million images, that we split into training, validation, and test sets with 314.460, 120.960, 48.420 images, respectively.

Standard models for image classification with different complexity like MobileNetV2 and VGG16, trained and tested with the SPMTH-60 dataset, do not perform particularly well. With the use of a combination of different strategies during training, including the reduction in a number of filters and the application of a strong image data augmentation, they can achieved a high accuracy in the classification of theoretical images, but failed dramatically when confronted with experimental AFM images. Our analysis of the activation of some filters in different blocks of the VGG16 architecture indicates that the model has run into overfitting: it has too many parameters and some of their filters in the deeper layers have specialized excessively during the training while others in the first ones remain completely random.

Standard models do not work with SPMTH-60 dataset as they are not suited to the grayscale information and to the small set of common features that dominate AFM molecular contrast. A properly designed model, like our ML-AFM, with the optimal depth and incorporating different paths for the data, provides the necessary flexibility to avoid overfitting and to achieve the training necessary to produce a successful classification. When posed with experimental images, ML-AFM performs rather well, but does not achieve the accuracy demonstrated with theoretical images. However, we have shown that it is possible to design a VAE to generate, from very few experimental images, a small set of AFM images to incorporate characteristic features of the experiments into our training dataset. The ML-AFM model, trained with this set composed mainly by theoretical images and enlarged with very few experiment-like images (just 0.17% of the total training set) is able to succeed with almost equal accuracy in the classification of both theoretical and experimental images.

Looking back at the theoretical and experimental AFM images shown in Figure 2 and Figure 3, it is possible to grasp the challenge that the molecular classification based on AFM images represents. We have shown that deep learning techniques provide a successful classification even in cases where it would be really hard for the human eye, such as discriminating among molecular structures that only differ in the nature of one halogen atom—extremely difficult to grasp even in purely theoretical AFM images—and coping with the large variations observed in experimental images of the same molecule, quite different in size, orientation, and internal contrast.

From this perspective, this work represents a promising step but we need to be aware of the problems ahead. Our classification has been restricted to 60 different molecular structures. If the model is confronted with an image that does not belong to any of the classes in the dataset used in the training, the output of the model will be still one of those 60 classes, and, thus, wrong. New strategies, probably based in the identification of the number and location within the molecule of atoms belonging to different chemical species, are needed to generalize the classification to cover the richness and complexity of organic chemistry.

## Figures and Tables

**Table 1:** Results achieved in the classification of 110 experimental images for acridine (ACR), carbazole (CAR), and dibenzothiophene (DIB) by the two standard models, MobileNetV2 (MNtV2) and VGG16, and the ML-AFM model developed in this work (see Section 3.2). Support refers to the number of images of each molecule included in the testing set. The column labelled “simulations” shows the results for the corresponding model trained only with theoretical simulations, while “VAE” corresponds to the results when the training set also includes the 540 images generated from only three experimental images with the VAE described in Section 3.3. This small enlargement, just 0.17%, improves significantly the performance of the standard models and confers our ML-AFM an accuracy closer to the one achieved with theoretical images.

		Simulations			VAE		
Molecule	Support	MNtV2	VGG16	MLAFM	MNtV2	VGG16	MLAFM
ACR	68	0.06	0.08	0.82	0.80	0.82	0.96
CAR	11	0.00	0.00	0.45	0.45	0.72	0.72
DIB	31	0.00	0.00	0.62	0.19	0.74	0.90

## Data Availability

All data published in this work are available upon reasonable request to the corresponding author.

## Abbreviations

The following abbreviations are used in this manuscript:

(NC)AFM	(non-contact) Atomic Force Microscopy
PPM	Probe Particle Model
FDBM	Full Density Based Model
VASP	Vienna Ab initio Simulation Package
DFT	Density Functional Theory
VAE	Variational Autoencoder
IDG	Image Data Generator

## Appendix A. Implementation and Training Details for the Deep Learning Models

Deep learning models have been implemented in Keras [67] running a Tensorflow backend [30]. In the case of the standard models, MobileNetV2 [45] and VGG16 [46], the first training was performed with transfer learning from ImageNet [68]. In order to allow the models to specialize in AFM imaging, we have trained both models with random starting weights. The results presented below for the loss and accuracy metrics of these models correspond to this second training.

### Appendix A.1. Image Data Generator

In order to avoid overfitting during the training of deep learning models, an image data augmentation is usually applied to the training set. This strategy has been employed for the training of all the models, MobileNetV2, VGG16, VAE, and our ML-AFM model. The image data generator (IDG) applies additional random deformations (like rotations, flip, zoom, shear, and shift) to the images in the training dataset. The application of this technique, that adds variation to the data used for the training in the different epochs, avoids overfitting and also provides versatility to reproduce or classify data that are quite different from the training dataset. Figure A1 shows the results of applying the IDG to a theoretical simulation of acridine.

We have tested several combinations of parameters for the IDG. All of them are useless in the MobileNetV2 and VGG16 cases but very effective in improving the results with both ML-AFM and the VAE. The best performing combination of parameters carried out in this work corresponds to the random choice of values in ranges of [−180,180] degree rotations, both horizontal and vertical flip, [−20,20]% for zoom, shear, and both width and height shift. When necessary, nearest filling has been applied for the points outside of the boundaries of the input. In this work, we have shown that, with the right choice of parameters for these deformations, ML-AFM obtains high accuracy in test with experimental images and that VAE is able to learn characteristic features from the experimental images that are absent from the theoretical simulations.

To check that the classification results are not a statistical fluke, we have performed three training runs of each model applying the IDG, randomly initializing the weights in all of them, and obtaining similar results in each test.

### Appendix A.2. MobileNetV2

Figure A2 shows the loss and accuracy metrics during the training of MobileNetV2 with halved filters. The training loss function has an extremely fast decrease in the first epochs reaching the value $10^{-2}$ at epoch 17, and decreasing smoothly from this point until almost reaching zero. In parallel, the accuracy metric has an extremely high growth in the firsts epochs, achieving an accuracy of 0.99 at epoch 17 in the test suite. From this epoch onwards, the training loss function decreases but the test accuracy does not improve, a clear indication that we should not train MobileNetV2 for more than 17 epochs. The graphs indicate that the model can still learn but also that it has reached maximum accuracy. These results show that the SPMTH-60 dataset is not complex enough to adjust the excessive amount of parameters of MobileNetV2 for this issue. If we also consider the fact that it fails dramatically in the test carried out with the experimental images (see Table 1), we deduce that the model does not perform a proper classification.

### Appendix A.3. VGG16

Figure A3 shows the loss and accuracy metrics of VGG16 during the training. To understand why the value of the validation metrics is better than the training metrics, it should be noted that an IDG (see Figure A1) has been applied to the training set during the fitting process, making the data more complex and, consequently, more difficult to classify.

The loss function metrics show that the model continues improving its learning up to epoch 60, while the accuracy metric shows an improvement up to this epoch. These metrics also show that the accuracy of the model is almost unbeatable at epoch 60, so we stop the training at that point, where it seems that the model is ready to perform an AFM classification. However, as explained in the main text, the model has the ability to learn more details, i.e., the data with which we have fed the model during the training have not been sufficiently varied for the model to update all its layers in the classification. This leads to a specialization of the model on theoretical images and, consequently, to poor results in the classification of AFM experimental images (See Table 1).

In order to visualize the transition of information between different layers, we show different kernels and activations of the VGG16 model on AFM images of benzene and anthracene. Figure A4 shows how the first convolutional layers *“let through"* almost all information while the information that reaches the deeper layers is so encoded that it is only readable by the model. It should be noted that the information in Figure A4 is not strictly sequential, i.e., the input of each convolutional layer is the output of each of the filters of the previous layers (from tens to hundreds). In order to reflect the data in a readable table we have selected a single filter from each convolutional layer and a single output for the selected filter.

### Appendix A.4. Our ML–AFM Model

The analysis of standard models has indicated that the difficulty of classifying experimental AFM images by training a model with theoretical simulations lies in designing a model that has an optimal depth to be able to generalize. The development of the ML–AFM architecture is inspired by this idea, with the goal of giving it, at the same time, sufficient depth, and enough regularization to avoid overfitting. As discussed in the main text, when properly trained, ML-AFM is able to obtain a remarkable accuracy in the test with both theoretical and experimental images.

Table A1 shows the details of each layer of the ML-AFM model depicted graphically in Figure 5. To prevent the scenario that leads standard models to misclassify, ML-AFM has been developed with different depths of convolutional layers, ensuring that the gradient value of the backpropagation is non-zero when it reaches the first layers. With a similar aim, the model has been developed with different regularization techniques, such as dropout layers [41] or kernel regularizers in several convolutional layers (See Table A1 and Figure 5).

**Table A1:** Each line represents a layer of the model graphically represented in Figure 5. From left to right we have represented by columns the input dimensions, the type of layer, the kernel size, the output channels, the stride length of the convolution and pool layers, the L2 kernel regularizer, the activation function and the connections with previous layers. The following list of abbreviations for layer names has been applied: Convolution2D (CV), Average Pool (AvPl), Max Pool (MxPl), Dropout (Dr), Concatenate (Conc).

	Input	Operator	Kernel	OC	Stride	KR-L2	Act	Connected to
Block 1	$224^{2}\times 1$	Input	-	1	-	-	-	-
	$224^{2}\times 1$	${\mathrm{AvPl}}_{111}$	(2,2)	1	(1,1)	-	-	Input
	$224^{2}\times 1$	${\mathrm{CV}}_{121}$	(3,3)	31	(2,2)	-	ReLU	Input
	$112^{2}\times 32$	${\mathrm{Conc}}_{1}$	-	32	-	-	-	${\mathrm{AvPl}}_{111}$,${\mathrm{CV}}_{121}$
Block 2	$112^{2}\times 32$	${\mathrm{Dr}}_{2}$	0.2	32	-	-	-	${\mathrm{Conc}}_{1}$
	$112^{2}\times 32$	${\mathrm{CV}}_{2}$	(3,3)	32	(1,1)	-	ReLU	${\mathrm{Dr}}_{2}$
	$112^{2}\times 32$	${\mathrm{MxPl}}_{211}$	(2,2)	32	(1,1)	-	-	${\mathrm{CV}}_{2}$
	$112^{2}\times 32$	${\mathrm{CV}}_{221}$	(3,3)	32	(2,2)	0.01	ReLU	${\mathrm{CV}}_{2}$
	$56^{2}\times 64$	${\mathrm{Conc}}_{2}$	-	64	-	-	-	${\mathrm{MxPl}}_{211}$, ${\mathrm{CV}}_{221}$
Block 3	$56^{2}\times 64$	${\mathrm{Dr}}_{3}$	0.2	64	-	-	-	${\mathrm{Conc}}_{2}$
	$56^{2}\times 64$	${\mathrm{CV}}_{311}$	(1,1)	64	(1,1)	0.01	ReLU	${\mathrm{Dr}}_{3}$
	$56^{2}\times 64$	${\mathrm{CV}}_{312}$	(7,1)	64	(1,1)	0.02	ReLU	${\mathrm{CV}}_{311}$
	$54^{2}\times 64$	${\mathrm{CV}}_{313}$	(1,7)	64	(1,1)	-	ReLU	${\mathrm{CV}}_{312}$
	$56^{2}\times 64$	${\mathrm{CV}}_{314}$	(3,3)	64	(1,1)	-	ReLU	${\mathrm{CV}}_{313}$
	$56^{2}\times 64$	${\mathrm{CV}}_{321}$	(3,3)	64	(1,1)	0.01	ReLU	${\mathrm{Dr}}_{3}$
	$54^{2}\times 64$	${\mathrm{CV}}_{322}$	(3,3)	64	(1,1)	-	-	${\mathrm{CV}}_{321}$
	$54^{2}\times 128$	${\mathrm{Conc}}_{3}$	-	128	-	-	-	${\mathrm{CV}}_{322}$,${\mathrm{CV}}_{314}$
Block 4	$54^{2}\times 128$	${\mathrm{Dr}}_{4}$	0.2	128	-	-	-	${\mathrm{Conc}}_{3}$
	$54^{2}\times 128$	${\mathrm{CV}}_{4}$	(3,3)	128	(2,2)	-	ReLU	${\mathrm{Dr}}_{4}$
	$27^{2}\times 128$	${\mathrm{AvPl}}_{4}$	(3,3)	128	(2,2)	-	-	${\mathrm{CV}}_{4}$
	$14^{2}\times 128$	${\mathrm{CV}}_{411}$	(1,1)	128	(1,1)	0.01	ReLU	${\mathrm{AvPl}}_{4}$
	$14^{2}\times 128$	${\mathrm{CV}}_{412}$	(3,3)	128	(1,1)	0.01	ReLU	${\mathrm{CV}}_{411}$
	$14^{2}\times 128$	${\mathrm{CV}}_{413}$	(3,3)	128	(2,2)		ReLU	${\mathrm{CV}}_{412}$
	$14^{2}\times 128$	${\mathrm{CV}}_{421}$	(1,1)	128	(1,1)	0.01	ReLU	${\mathrm{AvPl}}_{4}$
	$14^{2}\times 128$	${\mathrm{CV}}_{422}$	(3,3)	128	(2,2)	-	-	${\mathrm{CV}}_{421}$
	$7^{2}\times 256$	${\mathrm{Conc}}_{4}$	-	256	-	-	-	${\mathrm{CV}}_{413}$, ${\mathrm{CV}}_{422}$
Block 5	$7^{2}\times 256$	${\mathrm{CV}}_{5}$	(3,3)	256	(2,2)	-	ReLU	${\mathrm{Conc}}_{4}$
	$3^{2}\times 256$	${\mathrm{Dr}}_{5}$	0.2	256	-	-	-	${\mathrm{CV}}_{5}$
	$3^{2}\times 256$	Flatten	-	2304	-	-	-	${\mathrm{Dr}}_{5}$
	2304	FC	-	60	-	-	Softmax	Flatten

On the other hand, ML-AFM requires sufficient capacity to detect the different characteristics of each AFM image. This can be achieved with depth. Based on the analysis developed for VGG16 filters (Figure 4), we deduce that at least nine (and a maximum of 12) convolutional layers have been updated during the training. Therefore, we implement different model paths with these depths (Table A1). Each convolutional layer contains several kernels that are specialized in the detection of a particular feature. Since the problem we are dealing with is a grayscale image classification, AFM-model has few filters in each convolutional layer (Table A1) comparing it with other models that have been developed to perform three-channel image classification.

The effects of developing a specific model for SPMTH-60 are shown in Figure A5, where, compared to the metrics of the other two models, ML-AFM has greater difficulty in learning. This may seem a departure from our goal, but actually reflects that the model is not specializing too much in the training data and is going to be able to generalize, for example, to include the novel features appearing in the experimental images. The results of Table 1 confirm this idea: while VGG16 and MobileNetV2 provide essentially random results, ML-AFM is able to perform a reasonable classification, even when trained only with theoretical images.

### Appendix A.5. Variational Autoencoder

We have developed our VAE as a multilayer perceptron (MLP) with a latent space of dimension 3. The encoder network is composed by a flatten input layer with 50,176 units followed by five hidden fully connected layers with 4096, 1024, 256, 64, and 8 units each one. At this point the architecture is divided into two branches, both of them composed by a fully connected layer with 3 units each one, that are applied as mean and variance of a normal distribution in latent space as in the standard VAE [48]. The decoder is composed by six fully connected layers with 8, 64, 256, 1024, 4096, and 50,176 units, respectively, activated with ReLU [69] function except the last one that is activated with a sigmoid function. The VAE has been trained applying the IDG (Appendix A.1) to each input and applied Nadam [70] optimizer with keras default parameters.
